# Pain management in a patient with intestinal failure in the palliative care setting: case report

**DOI:** 10.1186/s12904-025-01878-0

**Published:** 2025-09-30

**Authors:** Adarsh Das, Jayamangala Sampath Kondasinghe

**Affiliations:** 1 Palliative Care Service, Sir Charles Gairdner Hospital, 1 Hospital Avenue, Nedlands, WA 6009 Australia; 2 Department of Palliative Care, Fiona Stanley Hospital, 11 Robin Warren Drive, Murdoch, WA 6150 Australia; 3 Internal Medicine, Faculty of Health and Medical Services, The University of Western Australia, 35 Stirling Highway, Crawley, WA 6009 Australia

**Keywords:** Gastrointestinal, Hypoganglionosis, Intestinal failure, Pain, Palliative care

## Abstract

**Background:**

Managing pain in patients with incurable intestinal failure requires a multidisciplinary approach that addresses complex pharmacological and systemic challenges while navigating prognostic uncertainties.

**Case presentation:**

This case report of gastrointestinal hypoganglionosis demonstrates the use of subcutaneous ketamine when conventional opioids fail due to intestinal malabsorption and dysmotility. Effective care required interdisciplinary collaboration, yet persistent discordance between the patient’s curative expectations and her life-limiting prognosis hindered timely advance care planning and community support transitions.

**Conclusions:**

Prognostic uncertainty, compounded by the condition’s rarity, highlights the need for early palliative care integration in non-malignant conditions, to ensure that care goals align with patient and family priorities. The case report advocates for adaptable care models that bridge inpatient and community services, even amid prognostic ambiguity, to prevent fragmented care during critical transitions.

## Background

Palliative care for patients with chronic, non-cancer conditions present unique challenges that differ from traditional cancer-focused palliative care [1, 2]. In particular, the management of pain in patients with intestinal failure, encompasses multifaceted clinical, psychosocial, and systemic considerations that test the boundaries of conventional palliative approaches [3].

Adult hypoganglionosis is an acquired, rare and complex condition that affects the enteric nervous system, resulting in gastrointestinal dysfunction and associated pain [4]. The disease is characterised by a reduction in the number of ganglion cells in the myenteric and submucosal plexuses of the intestinal wall, leading to impaired intestinal motility and function [4–6]. It is considered a subset of intestinal innervation disorders, accounting for approximately 3% to 5% of all such classified conditions [4–6]. Clinical features include chronic constipation, recurrent abdominal pain, distension, nausea, vomiting and intestinal obstruction [5, 7]. Diagnosis itself can be challenging due to the rarity and non-specific nature of its symptoms, which include endoscopic evaluation and full-thickness intestinal biopsies for histological examination [4, 7]. The pain associated with hypoganglionosis results from several factors: a) chronic intestinal distension due to impaired motility, b) intermittent intestinal obstruction, c) intestinal wall inflammation, and d) visceral hypersensitivity [4, 6, 7].

This case report illustrates the interplay between complex pain management and prognostic dilemmas in a patient with acquired gastrointestinal hypoganglionosis and includes a narrative review of pain management in intestinal failure and the role of palliative care service delivery models in chronic, non-cancer diseases.

## Case presentation

### Key aspects of history

A patient in her late 20 s, who since 2020, had multiple hospital admissions for recurrent small bowel obstructions [SBO] presenting as undifferentiated abdominal pain. She had no significant past medical or surgical history. In June 2022, after her eight hospital admission in under two years, she underwent a right hemicolectomy due to a caecal volvulus. Subsequent histopathology led to the diagnosis of gastrointestinal hypoganglionosis in the terminal ileum and caecum. During this admission, given she was unable to tolerate enteral feeding post-operatively, she was commenced on total parenteral nutrition [TPN] of three litres daily via a tunnelled small bore central venous catheter [CVC]. She was subsequently discharged home, with close outpatient follow up by her gastroenterologist and her multidisciplinary Intestinal Failure [IF] team.

In January 2023, she was diagnosed with stage four endometriosis. During her admission between March 2023 till September 2023, she underwent multiple peripherally inserted central catheter [PICC] insertions in her upper and lower limbs for prolonged courses of intravenous hydration, antibiotics, parenteral analgesia and anti-emetics. Her analgesia was managed by the Acute Pain Service [APS] team during this and subsequent admissions. She was trialled on the following medications: sublingual buprenorphine 200 µg to 400 µg pro re nata [PRN] four-hourly, oral tramadol 50 mg to 100 mg PRN four-hourly, topical buprenorphine patches at a weekly dose of 5 µg/hour [to a maximum weekly dose of 10 µg/hour], topical fentanyl patches at a maximum weekly dose of 12 µg/hour and oral tapentadol sustained release at a maximum dose of 50 mg twice a day. The maximum doses of these analgesic regimens were not reached due to inadequate abdominal pain relief, prompting frequent changes to her analgesia plan. Additionally, she did not perceive any benefit from topical or oral analgesia formulations, which often led to medication noncompliance. Eventually, short courses of intravenous ketamine infusions were identified as the only regimen effective in alleviating her abdominal pain. She was frequently discharged home following the infusions without any additional background or PRN analgesia. The pain team scheduled outpatient appointments to monitor her analgesia control, but she was unable to attend the three appointments that were provided. During an extended admission from November 2023 to February 2024, she underwent a significant surgical procedure involving exploratory laparotomy, adhesiolysis, ileo-colic resection, and end-ileostomy, which was complicated by a superior mesenteric vein and a PICC-line associated thrombosis. Due to her significant medical comorbidities, she experienced a rapid functional decline, rendering her wheelchair bound. Her mobility was limited to 15 m due to fatigue, and her weight in August 2024 was recorded at 32 kg, representing a 45% reduction from her last documented healthy weight in July 2020.

### Palliative care involvement

**Fig. 1:**
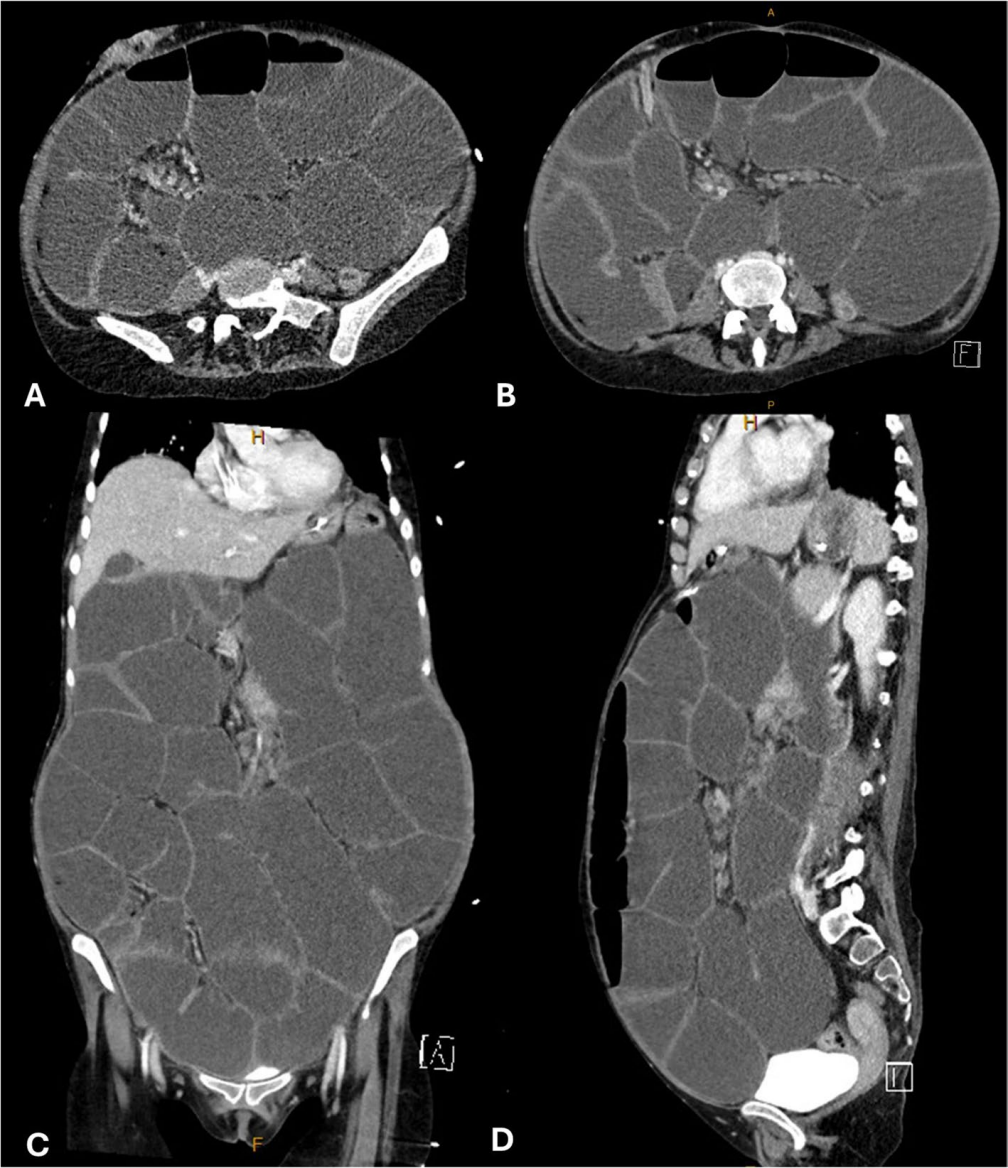
Abdominal Computed Tomography (CT) showing gastrointestinal ganglionosis with superimposed bowel obstruction in transverse (**A** and **B**), coronal (**C**) and sagittal views (**D**)

The palliative care [PC] team was first consulted during an admission in August 2024 for a partial small bowel obstruction [SBO] (Fig. 1). Her main presenting complaint was colicky, abdominal pain with spasms, which differed from the cyclical pain previously associated with her endometriosis. Physical examination showed a distended abdomen with diffuse tenderness and reduced bowel sounds. Based on the imaging and clinical findings, a nasogastric tube and foley catheter into her ileostomy were inserted for gastric decompression, and APS were referred to for pain management. Due to concerns about gastric absorption for oral medications given her partial SBO and previous ineffectiveness of sublingual buprenorphine, analgesia was strictly limited to parenteral. Initial attempts with intravenous tramadol, clonidine, and fentanyl provided limited relief to her abdominal pain. Eventually, an intravenous ketamine infusion of 2 mg/hour was effective, with adjuvant intravenous clonidine of 25 µg three-times a day. The ketamine infusion rate was set lower than standard protocols, and a loading dose was omitted. This adjustment was made due to concerns of opioid toxicity and polypharmacy, as the patient simultaneously was also being weaned off her intravenous fentanyl infusion. Her ketamine infusion was gradually titrated up to 8 mg/hour over two days without causing haemodynamic instability or hallucinations. On the fifth day of the ketamine infusion, her PICC line was blocked. Due to the previous PICC associated thromboses she had obtained in her contralateral limbs, no other long-term intravenous medication access options were available, as her tunnelled CVC was reserved for TPN. The APS subsequently consulted with the in-patients’ PC team to discuss alternative analgesia options. Analgesia was transitioned to a continuous subcutaneous infusion [CSCI] of 200 mg ketamine over 24 h. Intravenous clonidine was changed to a subcutaneous regimen of 25 µg administered three-times a day. As her SBO resolved, the ketamine administered via CSCI was gradually tapered from 200 to 150 mg after 24 h, which was subsequently reduced to 75 mg after 7 days. After a further 24 h, the CSCI was transitioned to just oral ketamine lozenges 25 mg to 50 mg PRN six-hourly. Given her reduced analgesia requirements, improving bowel obstruction and opioid toxicity concerns given her low body weight, second-line analgesia of subcutaneous hydromorphone was commenced at a low dose of 0.2 mg to 0.4 mg PRN one-hourly, without any additional background analgesia.

Despite the initial referral to palliative care indicating her prognosis of a few months, the patient and her family did not accept this prognosis. Her mother sought second opinions from private gastroenterologists for pursual of active treatment. Due to such discordance, gastroenterology and palliative care endeavoured to address prognosis and advance care planning, including limitations on high-level interventions, such as surgery and intensive care. However, the patient and her family consistently maintained that her condition was not a life-limiting illness. As her condition stabilised, the PC team encountered challenges in her discharge planning and had to address the constraints of community palliative care services for a patient who was unwilling to acknowledge that her disease was life-limiting. The community team, considering these premises and her pursuit of active treatment, initially could not accept the patient for providing community medical and nursing support for sublingual ketamine and parenteral hydromorphone. Efforts were made to re-engage APS and Chronic Pain Service [CPS] to establish a collaborative care model within the community. However, they were hesitant to re-engage due to her previous non-compliance with CPS outpatient clinics.

Despite the lack of engagement from the APS and CPS teams, and notwithstanding the patient’s denial concerning her prognosis, the inpatient palliative care team were able to arrange for the community PC service to provide clinical support upon discharge. She was discharged home after 50 days as an inpatient, with intravenous cyclizine 50 mg three times a day and daily TPN administered via her tunnelled CVC, without the need for background nor intermittent analgesia. Despite these efforts, the patient was found in septic shock from an infected tunnelled CVC line by the community PC team and was readmitted to the gastroenterology inpatient unit within 24 h of discharge. A goals of care discussion was held with the patient and her family by her gastroenterologist and the hospital intensivist to determine the appropriateness of intensive care admissions and rapid response interventions. After thorough consideration for her quality of life, it was agreed that these measures would not be beneficial. The medical ceiling of care was for ward-based treatment for reversible causes, which included the removal of her tunnelled CVC line, which she agreed to have removed. Whilst waiting for her procedure, the patient suffered a sudden cardiac arrest on the ward and died shortly after, with her family by her side, within 36 h of her discharge from the same hospital.

## Discussion

### Challenges in pain management for intestinal failure patients

Intestinal failure [IF], characterised by the inability to maintain adequate nutrition or fluid-electrolyte balance without parenteral support, often results in chronic visceral pain due to structural and functional gut abnormalities [8, 9]. Inflammatory changes, bacterial overgrowth and dysmotility contribute to nociceptive and neuropathic pain mechanisms [10, 11]. The reliance on parenteral nutrition introduces additional risks, including intravenous catheter infections, and intestinal failure associated liver disease, which exacerbate discomfort [9]. Opioids remain a cornerstone of analgesia management in IF but are fraught with challenges. Prolonged use is linked to opioid-induced hyperalgesia, dependency, and its potential to exacerbate gut dysmotility [10, 12, 13]. Oral opioid analgesics are often ineffective due to intestinal absorption challenges, necessitating intravenous, sublingual or transdermal routes [8, 10]. Addition of adjuvants such as gabapentin can address neuropathic components whilst minimising dependency, but circles back to lack of gut absorption through the oral route [14, 15]. Notably for our patient, gabapentin or pregabalin were not trialled due to concerns of gastrointestinal malabsorption; however parenteral clonidine was proven to be an effective analgesia adjunct.

Transdermal options such as buprenorphine and fentanyl patches are widely used for chronic visceral pain in IF. Buprenorphine’s partial μ-opioid agonism and κ-antagonism reduce constipation risk compared to full opioid agonists [16]. Transdermal drugs such as fentanyl and buprenorphine patches provide steady analgesia but require intact dermal perfusion, which may be compromised in malnourished and cachectic patients [17]. Sublingual buprenorphine and fentanyl avoid intestinal absorption issues in IF patients, and avoid inconsistent absorption from jejunostomies or ileostomies, providing rapid relief during acute pain flares [16, 17]. However, limitations include worsening of ileus and motility disorders (especially in patients with short gut syndrome), bacterial overgrowth, opioid dependency and regulatory restrictions in Australia on opioid naïve patients, particularly with fentanyl [10, 16, 17]. Furthermore, 30% of patients develop erythema and pruritus at the application sites for transdermal formulations, which complicates long term use [16, 17]. Recent advancements in pain relief for IF patients include teduglutide, a glucagon-like peptide-2 [GLP-2] analogue that enhances intestinal adaptation by increasing villus height and crypt depth, and reduces abdominal pain by mitigating mucosal inflammation [18]; eluxadoline, a peripherally acting μ-opioid agonist/δ-antagonist that reduces visceral hypersensitivity in diarrhea-predominant conditions [15]; and low dose transdermal clonidine which reduces colonic hypermotility and visceral pain via α_2_-adrenergic modulation [19]. However, high costs, accessibility barriers and risk of biliary complications prevent from mainstream use.

Intervention strategies such as coeliac plexus neurolysis and splanchnic nerve blocks have been considered for specific patient populations. Coeliac plexus neurolysis targets sympathetic nerves innervating the upper abdominal viscera. In IF patients with chronic pancreatitis or radiation enteritis as the predominant feature of their abdominal pain, coeliac plexus blocks reduced pain scores by 50–70% for three to six months [20]. Similarly, by targeting splanchnic nerves near the T11 – T12 vertebrae, this block is preferred for patients with retroperitoneal fibrosis or extensive abdominal adhesions [21]. Although not extensively studied, long term epidural or intrathecal blocks and other implanted neuraxial devices are generally considered unsuitable for managing pain related to IF. This is due to the higher risk of autonomic instability, potential motor blockade which could exacerbate muscle atrophy in malnourished patients and, and higher rates of catheter-related infections due to their immunocompromised status [22]. Non-pharmacological interventions, such as cognitive-behavioural therapy and acupuncture are underutilised despite evidence supporting their role in reducing opioid reliance [23, 24].

### Interdisciplinary care coordination and service delivery models

Given our patient’s complex needs, multidisciplinary coordination amongst specialties, including gastroenterology, surgery, APS, CPS and palliative care was required. Multiple studies demonstrate that multidisciplinary teams significantly improve pain control and quality of life in cancer patients. A prospective study conducted in China of 92 cancer inpatients found that individualised interventions by an interprofessional team consisting of surgeons, interventional radiologists, palliative care physicians, pain physicians, psychologists, nutritionists and nurses led to a reduced pain burden and pain scores, for patients receiving concurrent chemotherapy [25]. Notably, an Australian mixed-methods observational study demonstrated that patients participating in a chronic pain program with structured phone follow-up experienced a 44% reduction in annual hospitalisations, highlighting the critical role of continuity of care [26].

Collaboration between palliative care and pain medicine specialists is most effective when embedded within joint clinical frameworks. A survey of palliative care physicians found that institutions with dedicated collaboration systems and regular case conferences reported 2.5 times higher referral rates to pain medicine specialists, and co-managed 37% of cancer pain cases compared to 6% in less collaborative environments [27]. Despite physicians acknowledging the importance of their respective role in patients’ care, key barriers to referrals in non-collaborative systems include role ambiguity, lack of funding for interdisciplinary time (which in turn disincentivises collaboration) and insufficient time for intricate discussions [28–30]. Successful collaborative palliative care service models include a combination of hospital-based consult teams and community-based palliative care. Inpatient palliative care teams reduce intensive care unit admissions by 25% through early symptom management and discharge planning, whereas community palliative care programs reduce hospitalisation rates by almost 40% [31, 32]. A more collaborative interdisciplinary care coordination with the in-patient and community service models might have helped avoid our patient’s unsuccessful discharge to the community and the limited time spent there before her final admission, which ultimately led to her demise. Notably she was not linked to any rare disease communities such as “Rare Voices”, which, through their capacity building and authentic engagement, recognise shared healthcare experiences of rare diseases and offer practical examples to address patient and family needs [33].

### The role of palliative care in non-malignant conditions

Non-cancer conditions such as chronic obstructive pulmonary disease [COPD], end-stage congestive cardiac failure and dementia account for 60% of people with palliative care needs, but only 14% of specialist palliative care referrals [34, 35]. Prognostic uncertainty, compounded by variable disease trajectories, causes delays in referrals, as clinicians hesitate to label patients as “palliative” [35, 36]. This is exemplified in this case, where the patient and her family were in-denial of her life-limiting disease, given the rarity of gastrointestinal hypoganglionosis, and its limited data on long-term outcomes [5]. Tools like the surprise question ("Would I be surprised if this patient died in the next year?") and the Supportive and Palliative Care Indicators Tool [SPICT] improve identification but lack sensitivity in early-stage illness [32, 36]. Early palliative care integration, concurrent with disease-modifying treatments, enhances quality of life. For example, COPD patients receiving earlier palliative care report 30% fewer hospitalisations and improved dyspnoea management, via prioritising the relief of burdensome symptoms such as dyspnoea, fatigue and pain over prognosis [36, 37]. Strategies involving early goals of care discussions and facilitating early advance care planning reduces intensive care unit admissions by 50% in geriatric populations with advanced heart failure [38]. The “Bowtie model”, as described by Hawley in 2014, presents an integrative framework that combines palliative care with chronic disease management from the point of diagnosis in patients with serious illnesses [39]. This approach aims to facilitate earlier acceptance of palliative care, irrespective of the prognosis or severity of the diagnosis. Embracing uncertainty and adopting early integration of palliative care principles at the onset of diagnosis may have yielded a different and more comfortable outcome for the patient and her family.

### Limitations

There are a few limitations in this case report. Methadone was not trialled as an option due to the patient’s non-compliance with chronic pain outpatient services and medication non-adherence. Recognised for its transmucosal and oral absorption characteristics as well as its NMDA receptor antagonism, methadone may be useful for patients with intestinal failure and for reducing opioid tolerance in those that require long term opioid therapy with an uncertain life expectancy [40–42]. Additionally, the intravenous ketamine dosing did not follow standard protocols described in the literature; however, this compromise was intentionally done to prioritise patient safety [43].

## Conclusion

This case study of a young woman with intestinal failure due to hypoganglionosis provides several important insights into palliative care: challenges in managing pain in intestinal failure; need for flexibility in life-limiting conditions with uncertain prognosis; and importance of interdisciplinary care coordination when treating complex patients.

## Data Availability

No datasets were generated or analysed during the current study.

## Abbreviations

SBO: Small bowel obstruction
TPN: Total parenteral nutrition
CVC: Central venous catheter
IF: Intestinal Failure
PICC: Peripherally inserted central catheter
APS: Acute Pain Service
PRN: Pro re nata
PC: Palliative care
CSCI: Continuous subcutaneous infusion
CPS: Chronic Pain Service
COPD: Chronic obstructive pulmonary disease
SPICT: Supportive and Palliative Care Indicators Tool
